# Homochiral nanotubes from heterochiral lipid mixtures: a shorter alkyl chain dominated chiral self-assembly†
†Electronic supplementary information (ESI) available. See DOI: 10.1039/c9sc00215d


**DOI:** 10.1039/c9sc00215d

**Published:** 2019-02-20

**Authors:** Xuefeng Zhu, Yuqian Jiang‡, Dong Yang, Li Zhang, Yuangang Li, Minghua Liu

**Affiliations:** a Beijing National Laboratory for Molecular Science (BNLMS) , CAS Key Laboratory of Colloid Interface, and Chemical Thermodynamics , Institute of Chemistry , Chinese Academy of Sciences , Beijing 100190 , P. R. China . Email: liumh@iccas.ac.cn; b National Center for Nanoscience and Technology , Beijing 100190 , P. R. China

## Abstract

It is an important topic to achieve homochirality both at a molecular and supramolecular level. While it has long been regarded that “majority rule” guides the homochiral self-assembly from an enantiomer mixture, it still remains a big challenge to manipulate the global homochirality in a complex system containing chiral species that are not enantiomers. Here, we demonstrate a new example wherein homochiral nanotubes self-assembled from a mixture of heterochiral lipids that deviated from the “majority rule”. We have found that when two heterochiral lipids with mirror headgroups but a 2-methylene discrepancy in alkyl chain length are mixed, homochiral nanotubes are always formed regardless of their mixing ratio. Remarkably, the helicity of the nanotube is exclusively controlled by the molecular chirality of the lipids with shorter alkyl chains, *i.e.*, the chiral self-assembly was dominated by the lipid with the shorter alkyl chain. MD simulation reveals that the match of both the alkyl chain length and hydrogen-bonding between two kinds of lipids plays an important role in the assembly. This work provides a new insight into the supramolecular chirality of complex systems containing multi chiral species.

## Abstract 2

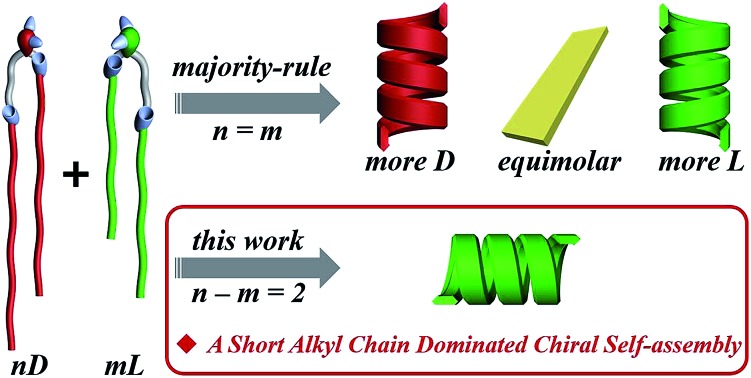
It is an important topic to achieve homochirality both at a molecular and supramolecular level.

## Introduction

Homochirality in living organisms, *i.e.* almost all of the amino acids and sugars are l- and d-enantiomers, respectively, is one of the most mysterious phenomena and has attracted long-term interest in biology, chemistry, physics and material science.^1^–^12^ Such molecular homochirality in the biological system requires the design of drug molecules as a single enantiomer,^13^ which is suggested to be related to the different interactions between proteins and enantiomers of drug molecules.^14^,^15^ Thus, the homochirality issue^16^–^21^ is extended to a supramolecular level such that the stereochemical communication or chiral–chiral interaction between various chiral species becomes vitally important.^22^–^25^ So far, two important rules on stereochemical communication, the “majority rule”^26^–^36^ and “sergeant-and-soldiers rule”,^27^,^31^,^37^–^42^ have been well-established with respect to covalent and non-covalent bonding of chiral polymers or supramolecular assemblies. Generally, the “majority rule” is related to two chiral molecules with mirrored configuration and states that the global chirality of the system is always determined by the chirality of the excess enantiomeric species. The “sergeants-and-soldiers rule” deals with the interaction between chiral sergeants and achiral soldiers and states that the chirality of the whole system follows the chirality of the sergeant. However, there is still a big challenge to manipulate the interaction or communication between different chiral species in complex systems,^3^,^43^–^48^ such as chiral lipids with different chain lengths in a biological membrane,^49^–^51^ where the chiral species are not necessarily in exact mirror configurations.^52^–^54^

**Fig. 1:**
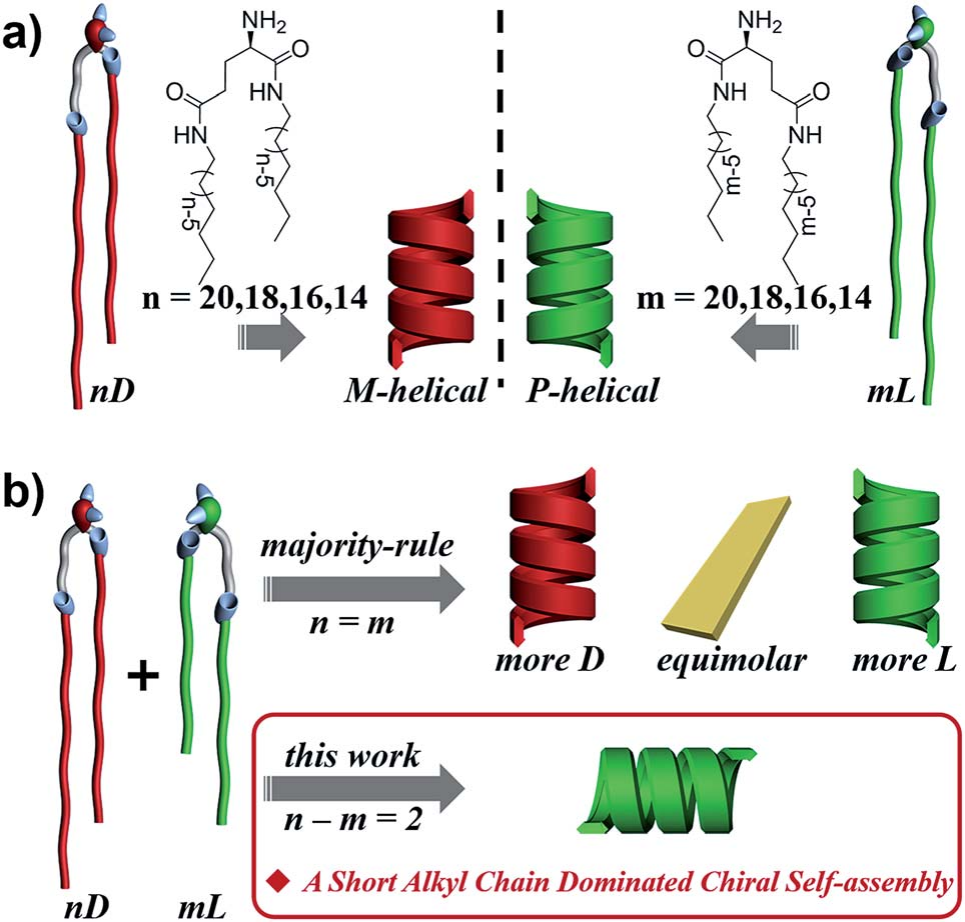
Self-assembly of chiral lipids. (a) Enantiomerically pure D- and L-lipids form M- and P-helices, respectively. (b) Mixing of racemates follows the “majority-rule”. However, mixing of two heterochiral lipids with mirror chiral head groups but a 2-methylene discrepancy in alkyl chain length leads to the homochiral composite nanotube, whose helical sense is exclusively determined by the molecular chirality of the lipid with the shorter alkyl chain regardless of their mixing ratios.

Here, we designed a series of enantiomeric glutamide lipids with various alkyl chain lengths and investigated their self-assembly behaviours (Fig. 1). Absolutely mirrored heterochiral lipid mixtures are found to follow the “majority rule”, *i.e.* the majority enantiomers control the global chirality of the system and the racemate is often achiral. However, when two heterochiral lipids with mirror headgroups but a 2-methylene discrepancy in alkyl chain length were mixed, a homochiral composite nanotube was always obtained. Remarkably, the helical sense was not determined by the majority component but by the lipids with the shorter alkyl chain no matter how small the amount of that lipid. This phenomenon deviates from the reported stereochemical communication rules and has never been reported before. It demonstrates that a small variation in molecular structure also plays an important role in stereochemical communication apart from intrinsic molecular chirality. By combining various experimental characterization methods and theoretical molecular dynamics (MD) simulation, the mechanism of this unprecedented phenomenon is disclosed.

## Results and discussion

### Lipid molecule design and synthesis

*N*,*N*′-bis(alkyl)-d/l-glutamic diamide lipids, with enantiomerically pure glutamic acid as the polar headgroup and double hydrophobic nonpolar alkyl tails, were designed to mimic natural amphiphilic chiral lipids with different chain lengths (Fig. 1). The lipid molecules were synthesized by two simple steps, as previously reported:^55^ the *tert*-butoxycarbonyl (Boc)-protected d/l-glutamic acid was firstly connected to two equimolar alkyl amines, then the Boc group was eliminated to free the polar amine headgroup.

### Self-assembly of heterochiral lipid mixtures

The self-assembly of the lipids was all performed in ethanol medium through a heat-and-cooling gelation process. Briefly, lipids or their mixtures were dispersed into ethanol at room temperature and then heated to a transparent solution. After the solution was cooled down to room temperature, the gel was formed. All the lipids as well as their mixtures could form white opaque gels and self-assembled into well-defined nanostructures upon gelation (see Experimental section for details).

### Characterization of the self-assembled nanostructures from heterochiral lipid mixtures

**Fig. 2:**
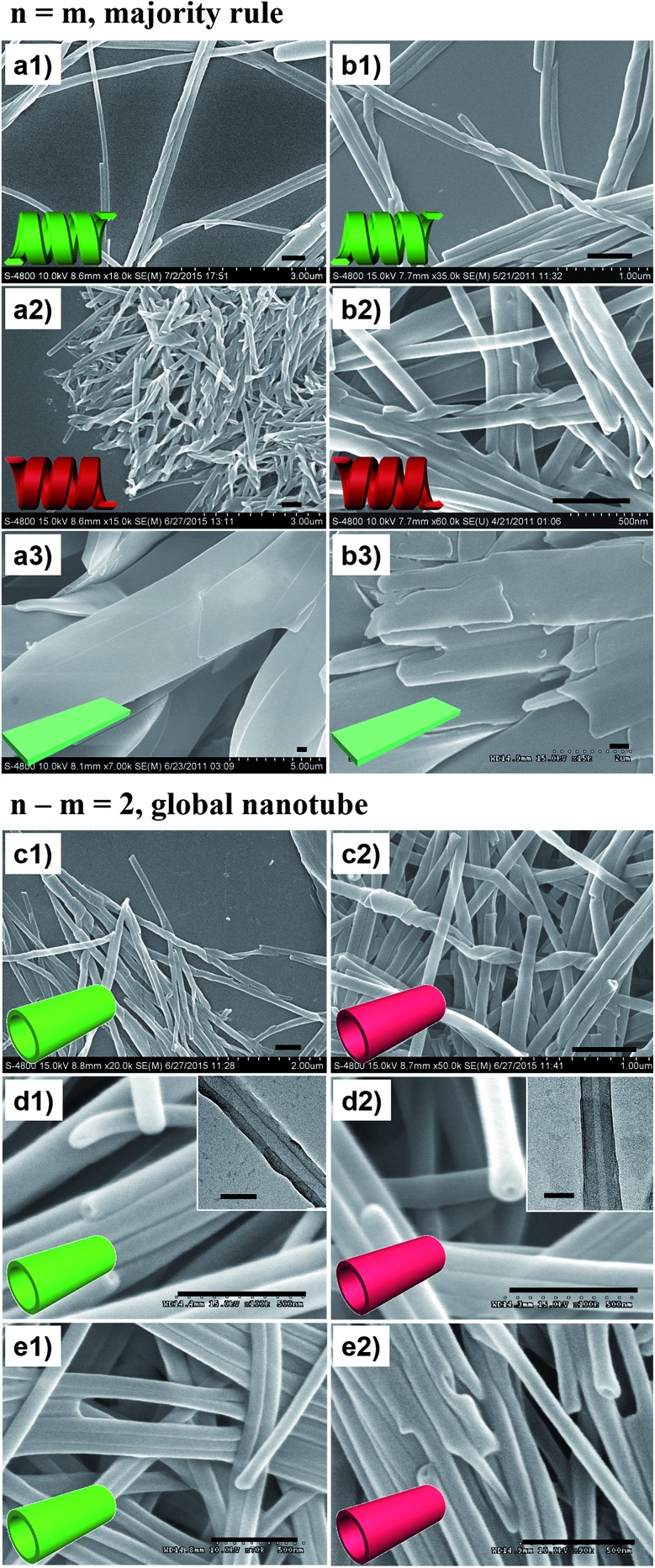
SEM morphologies. Majority rule for *n* = *m* heterochiral lipids: (a1) 20L, (a2) 20D, and (a3) 20D/20L = 1/1 (mol mol^–1^, the same below), and (b1) 16L, (b2) 16D, and (b3) 16D/16L = 1/1. Homochiral nanotubes formed from *n*–*m* = 2 heterochiral lipids at equimolar ratios: (c1) 20D/18L, (c2) 20L/18D, (d1) 18D/16L, (d2) 18L/16D, (e1) 16D/14L, and (e2) 16L/14D. Scale bar: 500 nm (SEM), 100 nm (TEM). Insets: cartoon illustration of morphologies. Fully aged gel (0.03 M in ethanol) was drop cast on single-crystal silicon wafers and carbon-coated Cu grids for SEM and TEM observation, respectively (the same below).

Fig. 2 shows the representative morphologies of the nanostructures, and three important features can be found.

First, all the pure enantiomers formed chiral nanotubes with helicity following their molecular chirality, regardless of their chain length, *i.e.*, D- and L-lipids produced left- and right-handed nanotubes, respectively (Fig. 2a and b).

Second, when two opposite enantiomeric lipids with absolute mirror-configuration (*n* = *m*) such as 20D/20L, 18D/18L, and 16D/16L were mixed, they obeyed the “majority rule”, *i.e.*, the helicity was determined by the excess enantiomeric lipid. In particular, a planar nanosheet without any chirality was formed for an equimolar mixture (Fig. 2a and b).

Third, when two pseudo-enantiomeric heterochiral lipids, *i.e.*, with opposite chiral head groups and a 2-methylene discrepancy in chain lengths, such as the combinations of 20L/18D, 20D/18L, 18D/16L, 18L/16D, 16L/14D, and 16D/14L, were mixed, helical nanotubes were exclusively formed at various mixing ratios, even for equimolar mixtures (Fig. 2c–e, S1 and S2†). In this case (*n*–*m* = 2 system), the “majority rule” is no longer operative.

In order to elucidate these new observations, various characterization methods, such as XRD, FTIR spectroscopy, CD spectroscopy and DSC thermal analysis, were carried out. Hereafter, the self-assembly of the 18D/18L and 18D/16L systems will be studied as an example.

**Fig. 3:**
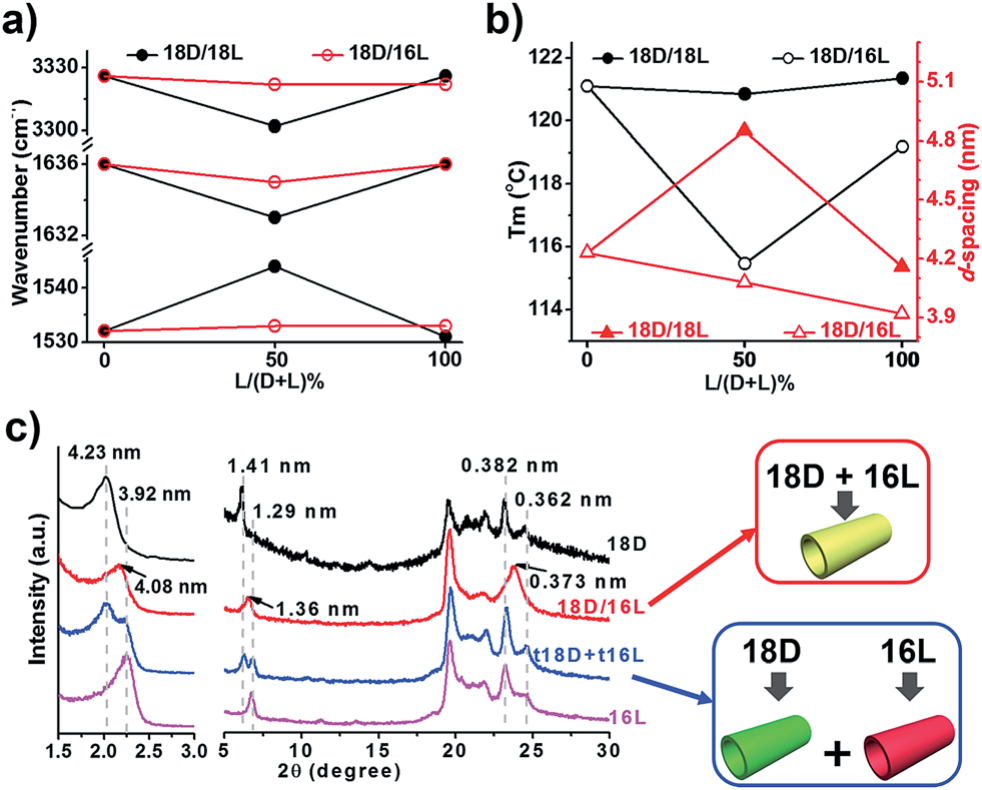
Correlative plots (a, vibration bands from FTIR spectra; b, *d*-spacing value from XRD and melting point from DSC) and XRD patterns (c) of self-assembled lipid nanostructures. 18D and 16L denote the nanotubes from the corresponding lipids; 18D/18L denotes the nanosheet from an equimolar mixture of 18D and 18L; 18D/16L denotes the composite nanotube from an equimolar mixture of 18D and 16L; t18D/t16L denotes a solid mixture of pre-self-assembled 18D nanotubes and 16L nanotubes at equal weight.

FTIR spectra are powerful in discriminating molecular interactions. As shown in the FT-IR spectra (Fig. S3†), all the nanostructures showed obvious H-bonded vibrations from N–H, amide I and amide II. However, their precise vibrations are different for the different lipid mixtures. The N–H, amide I and amide II bands at 3326, 1636, and 1531 cm^–1^ for the 18D (18L) nanotube shifted to 3302, 1633, and 1544 cm^–1^ for the 18D/18L nanosheet, indicating that the 18D/18L nanosheet has stronger hydrogen bonding interactions than that of either the 18D or 18L nanotube^55^ (Fig. 3a and S3, Table S1†). This was further confirmed by DSC thermogram analysis of the 18D/18L nanostructures (Fig. 3b and S4†), where the phase transition temperature (*T*_m_) is *ca.* 121 °C regardless of the mixing ratio, indicating the miscible nature of the 18D and 18L lipids.^56^ This means that the nanoscale chirality is counterbalanced at a molecular level.^55^ Consequently, the helical torsion force in the racemate bilayer is decreased, which is evidenced by the *d*-spacing expansion of the racemate bilayer (4.85 nm, equimolar 18D/18L) compared to the enantiomerically pure bilayers (4.23 nm, 18D) (Fig. 3c). Therefore, achiral planar nanosheets are produced for 18D/18L at an equimolar ratio.

In contrast, the FTIR spectra showed scarcely any change of the hydrogen bonding interaction in all 18D/16L combinations compared to the 18D or 16L nanotubes (Fig. 3a and S3, Table S2†). It seems that the helical torsion force in the heterochiral bilayer of 18D/16L is unaffected. Therefore, the nanotube rather than the planar sheet formed for all heterochiral 18D/16L combinations. However, only one *T*_m_ peak was found in the DSC thermograms of the 18D/16L nanotubes (Fig. S4†) and the plot of *T*_m_ value to mixing ratio is a U-shaped curve (Fig. 2b), with a *T*_m_ value of 115 °C for equimolar 18D/16L lower than those of either 18D (121 °C) or 16L (119 °C), suggesting the mutual diluent effect and co-self-assembly^30^ of 18D and 16L.^56^ Moreover, the XRD patterns (Fig. 3c) showed a single bilayer (4.08 nm) just between that of 18D (4.23 nm) and 16L (3.92 nm), further suggesting the co-assembly of all 18D/16L combinations. It should be noted that if two respectively self-assembled nanotubes were mixed, we can observe two sets of peaks (Fig. 3c). Therefore, we can conclude that when 18D and 16L were mixed, they tended to co-assemble rather than self-sort.

### Supramolecular chirality of the composite nanotubes from heterochiral lipid mixtures

**Fig. 4:**
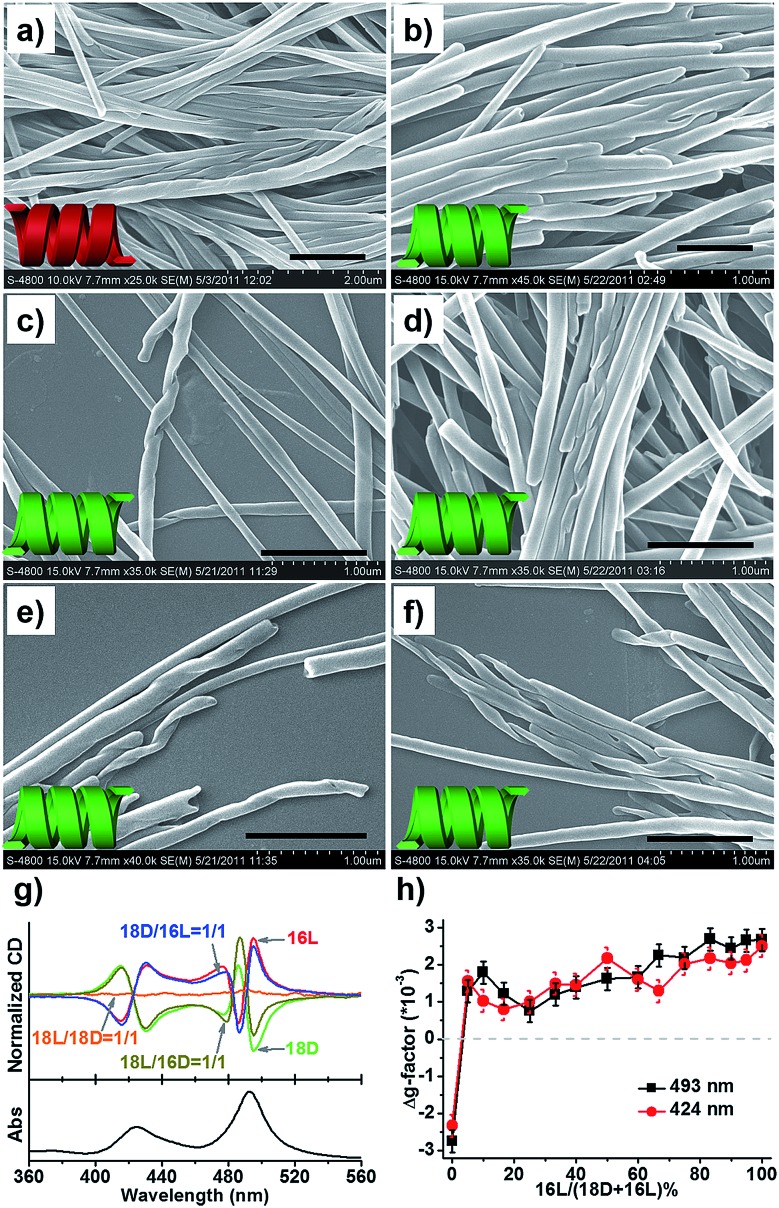
Supramolecular chirality of the self-assembled nanostructures from heterochiral lipids at various molar ratios. SEM images of (a) 18L/16D = 1/1, (b) 18D/16L = 1/1, (c) 18D/16L = 1/1.5, (d) 18D/16L = 1.5/1, (e) 18D/16L = 3/1, and (f) 18D/16L = 19/1. Scale bar: 1 μm. (g) CD spectra of the nanostructures with the TPPS probe. (h) Δ*g*-factor of the 18D/16L nanotube obtained with the TPPS probe at various molar ratios of 18D/16L at 493 and 424 nm.

Given that two opposite chiral lipids are involved in the *n*–*m* = 2 system, the supramolecular and nanoscale chirality of the composite nanotubes is alluring. High-resolution SEM images (Fig. 4a–f) show that the composite nanotube is chiral at the nanoscale. Moreover, the chirality is exclusively one handed, which is always consistent with that of the nanotubes formed from the shorter lipids alone. Specifically, the composite 18D/16L nanotubes are always right-handed (Fig. 4b–f) like the 16L nanotube and the 18L/16D nanotubes are left-handed (Fig. 4a and S5†) like the 16D nanotube. Obviously, the helicity of the composite nanotubes from heterochiral lipids is basically determined by the molecular chirality of the shorter lipids.

The helicity of the nanotubes was further investigated by CD spectroscopy. Since these lipid molecules do not possess any chromophore, an achiral dye, *meso*-tetra(4-sulfonatophenyl) porphyrin (TPPS), was used as a probe^57^–^59^ to reflect the helicity of the nanotube through aggregation on the surface of the nanotubes (Fig. 4g, h and S6†). UV/Vis spectra displayed two strong bands at 493 and 708 nm, indicating induced J-aggregation of TPPS at the surface of all nanotubes.^57^ The CD spectra of the D-lipids displayed two strong Cotton effects at 495(–) and 486(+) with a crossover at 490 nm, and 430(–) and 415 (+) with a crossover at 422 nm, while the L-lipids showed mirrored Cotton effects to those of the D-lipids, which reflected the chiral packing manner of the lipids at the surface of the nanotubes, *i.e.* an M-helix for D-lipids and P-helix for L-lipids. Both 18D/18L and 16D/16L were CD silent, indicating achiral packing at the surface of the planar nanosheets. On the other hand, 18L/16D and 18D/16L displayed strong negative and positive Cotton effects, respectively. Once 16L was involved in the system, 18D/16L exclusively showed positive CD signals regardless of the molar ratios of 16L to 18D (Fig. 4h). The CD results are well consistent with the SEM observations, indicating that the heterochiral lipid nanotubes are globally homochiral and that the helicity is essentially determined by the lipids with the shorter alkyl chain.

### Theoretical analysis and molecular dynamics (MD) simulation^60^–^63^

**Fig. 5:**
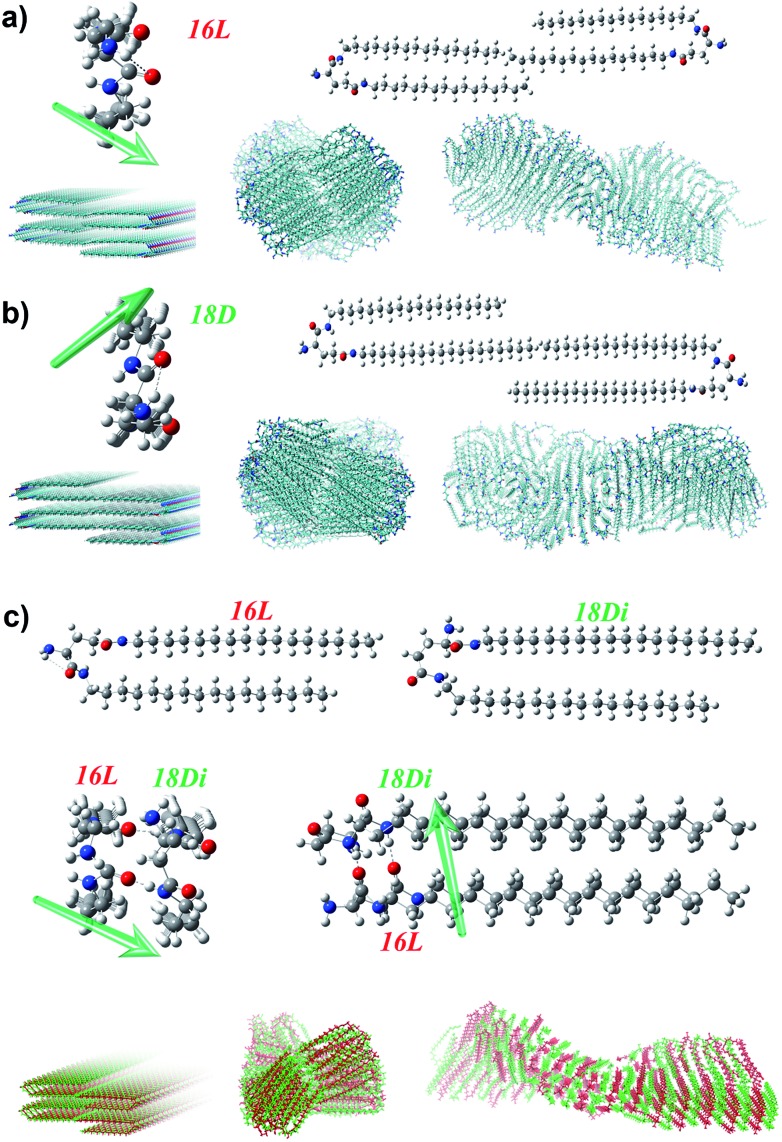
Calculation model and mechanism of the self-assembly of pure lipids (a, b) and heterochiral lipids (c). The intramolecular hydrogen bonding between α-amide and amino groups induces the α-amide to produce an orientation, and the opposite chirality leads to a *ca.* 90° difference in the directors (d, green arrow) of α-amide groups in 16L (a) and 18D (b). As for 18D/16L (c), the shorter lipid 16L induced a conformation rearrangement of the longer lipid 18D, leading to the disappearance of the orientation of the α-amide and an induced orientation of the γ-amide in 18D, which is the same as that of the α-amide in 16L. In this case, the alkyl chains between the two lipids are also perfectly matched. Therefore, a P-helical bilayer was achieved for the 18D/16L heterochiral lipid mixture like that of pure 16L.

To further disclose the unprecedented phenomenon and deeply understand the chiral self-assembly process, theoretical analysis was carried out *via* MD simulation. According to the previous theoretical studies,^64^,^65^ the handedness of aggregates is dependent on the molecular orientation, which is actually the orientation of amide groups in the lipid molecules here. Besides, the alkyl chain should be matched to maintain the bilayer. Therefore, we mainly focus on the alkyl chain length match and the orientation of amide groups to analyze the handedness of the heterochiral lipid bilayer. There are two amide groups in both 16L and 18D. The α-amide and amino groups can form an intramolecular hydrogen bond, which induces the α-amide to produce an orientation, while the direction of the γ-amide is uncertain. The interaction of the oppositely chiral headgroups leads to a *ca.* 90° difference in the directors (*d*) of the α-amide groups in 16L and 18D (Fig. 5a, b and S8†). The theoretical studies by Selinger *et al.* showed that rotating the tilt direction by 90° should change the curvature direction by 90°, giving a handedness reversal.^64^ Therefore, the different chirality of 16L and 18D bilayers can be easily understood.

Planar 16L (18D) bilayer aggregation has two different stacking manners (Fig. S9†). However, the aggregation with C2 symmetry (essentially the orientations of α-amides on two sides) where the rotation axis lies along the bilayer aggregation direction will lead to damage of the bilayer structure after MD simulation. Only the pre-assembly aggregation with C2 symmetry where the rotation axis lies perpendicular to the bilayer plane can result in chiral bilayer structures. For obtaining the chiral structures of the pure 16L and 18D systems, we built planar bilayer aggregates containing two layers and a total of 120 molecules with a 3.6 Å *d*-space for MD simulations. After the equilibriums were reached, we sampled one snapshot per 1 ps and extracted the average configurations during 5.5–6 ns for the pure 16L and 18D systems. It was found that the 16L molecules form a P-helix bilayer structure (Fig. 5a), while the 18D molecules form an M-helix (Fig. 5b). For the 16L/16D mixture with a 1 : 1 ratio, the lengths of alkyl tails perfectly match with each other and the intermolecular hydrogen bonds can form between α-amide groups. Moreover, the α-amide orientations of 16L and 16D are perpendicular to each other, finally resulting in the achiral nanosheet structure.

However, in the 16L/18D mixture, a conformation rearrangement on the molecular structure of 18D happened due to the existence of 16L. As presented in Fig. 5c, when the α-amide in 16L was connected to the γ-amide in 18D, and the γ-amide in 16L was connected to the α-amide in 18D, the length of the alkyl chains between the two molecules could be perfectly matched. In this situation, the orientation of the α-amide in 18D was lost, while the orientation of the γ-amide in 18D was induced and it pointed in the same direction as that of the α-amide in 16L. Hence, for further study on the 16L/18D aggregate by MD simulation, we built a pre-assembly bilayer with a planar structure containing two layers and a total of 120 molecules (16L/18D = 1/1). As with the pure systems, the orientations of the amides on both sides of 16L/18D should keep C2 symmetry where the rotation axis is perpendicular to the bilayer plane. After the equilibrium was reached, we also sampled one snapshot per 1 ps and extracted the average configuration during 5.5–6 ns for the 16L/18D system. It was found that a P-helix was achieved for the 16L/18D aggregate. The MD simulation is consistent with the experimental results, and well explains the unprecedented phenomenon.

## Conclusions

**Fig. 6:**
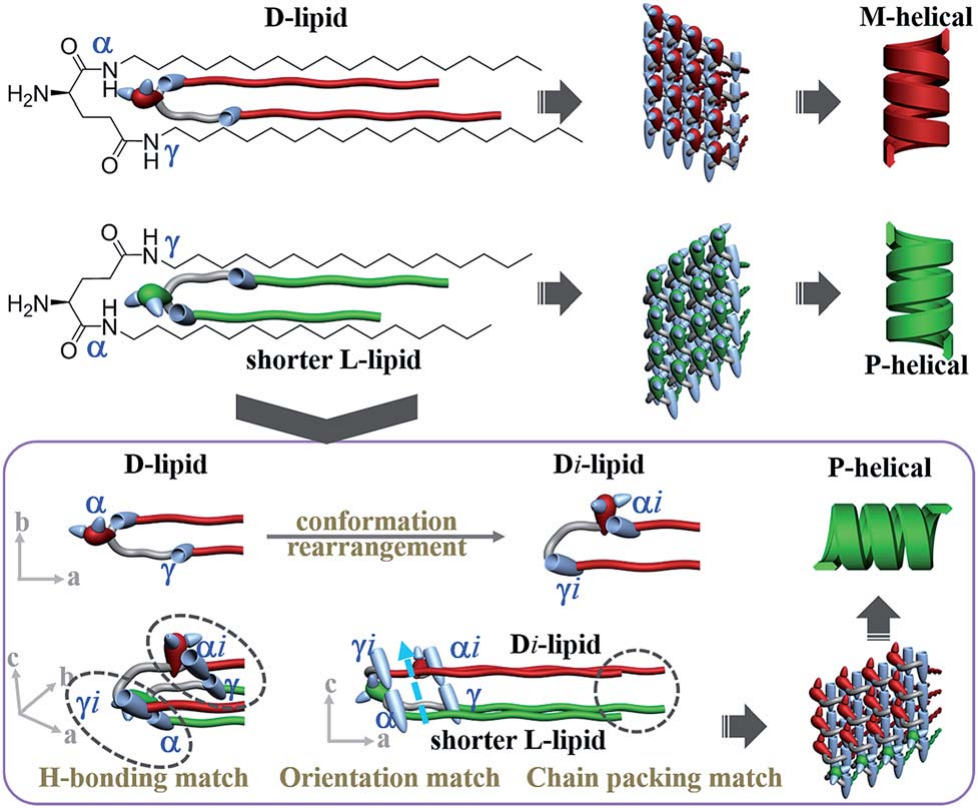
“Induced conformation rearrangement” mechanism of homochiral nanotube from heterochiral lipids. Alkyl chain communication between heterochiral lipids induced the conformation of the longer lipids to rearrange in the presence of the shorter lipids. Consequently, the orientation of the γ-amide of the longer lipids was induced and pointed in the same direction as that of the α-amide of the shorter lipids, while the orientation of the α-amide was lost. Finally, the alkyl chain packing, hydrogen-bonding connection and orientation of the two lipids were perfectly matched. Thus, globally homochiral nanotubes were produced and the helicity was exclusively determined by the molecular chirality of the shorter lipids.

In summary, the self-assembly behaviors of two heterochiral lipids and their mixtures were systematically investigated (Fig. 6). For individual chiral lipid self-assembly, the intramolecular hydrogen bond between the α-amide and amino groups induces the α-amide to produce an orientation, and the oppositely chiral headgroups cause a *ca.* 90° difference in the directors of the α-amide groups. Consequently, L-lipids always form P-helical nanotubes and D-lipids form M-helical nanotubes.

For the absolutely mirrored heterochiral lipid mixtures (*n* = *m* system), the lengths of the alkyl tails can perfectly match with each other and intermolecular hydrogen bonds can form between α-amide groups. In the composite bilayer, the α-amide orientations of the L-lipids and D-lipids are perpendicular to each other, finally resulting in the achiral nanosheet structure.

For the two heterochiral lipids with mirror headgroups but a 2-methylene discrepancy in alkyl chain length (*n*–*m* = 2 system), under alkyl chain communication, the conformation of the longer lipids is rearranged in order to match the shorter lipids (Fig. 6). Consequently, the α-amide of the short lipids was connected to the γ-amide of the longer lipids, and the γ-amide of the shorter lipids was connected to the α-amide of the longer lipids. In this situation, the alkyl chain length between the two lipids could be perfectly matched. Moreover, the orientation of the γ-amide of the longer lipids was induced and pointed in the same direction as that of the α-amide of the shorter lipids, while the orientation of the α-amide of the longer lipids was lost. Finally, the alkyl chain packing, hydrogen-bonding connection and orientation of the two lipids were perfectly matched. Thus, globally homochiral nanotubes are produced and the helicity of the heterochiral lipid nanotube is exclusively determined by the molecular chirality of the shorter lipids. The “induced conformation rearrangement” mechanism well interpreted the formation of the homochiral nanotube from heterochiral lipid mixtures regardless of the mixing ratio.

The present contribution sheds new light on the understanding of homochirality at a supramolecular and nanoscale level in complex lipid systems and provides new guidance in exploring homochiral materials in complex supramolecular systems.^3^,^43^–^45^

## Experimental

### Self-assembly procedure

For the self-assembly of pure lipids: the lipid solids (3 × 10^–5^ mol) were put into a seal-capped vial with 1 mL of ethanol added (0.03 M). Then, the sample vial was heated up to 75 °C for a while to make a clear solution and subsequently allowed to cool down to room temperature naturally (25 °C, cooling rate was about 10 °C min^–1^). White gels were obtained, which were fully aged for 12 hours under ambient conditions before being measured. For the self-assembly of mixed lipids: the required amount of D- and L-lipids was mixed at a specific proportion in one sample vial and 1 mL of ethanol was added (the total concentration was kept at 0.03 M). Then, the sample was treated using the above procedure.

### Characterization

#### SEM and TEM

The fully aged gel was transferred from a sample vial to single-crystal silica wafers with a thin film of Pt coating for SEM observation and to carbon-coated Cu grids stained with 2% uranyl acetate (wt%, aqueous, about 2 min) for TEM observation; images were taken using a Hitachi S-4300 or S-4800 FE-SEM (15 kV) and a JEM-2010 (200 kV), respectively.

#### XRD

The quartz-plate-sustained xerogel films of self-assembled lipids or lipid mixtures were used for XRD measurements on a Rigaku D/Max-2500 X-ray diffractometer (Japan) with Cu/Kα radiation (*λ* = 1.5406 Å, 40 kV, 200 mA). For t18D/t16L, two respectively pre-self-assembled nanotubes of 18D lipid and 16L lipid were vacuum-dried, and the solids were mixed and ground with an agate mortar and pestle for fully mixing.

#### FTIR spectroscopy

KBr pellets of vacuum-dried xerogels were prepared for Fourier-transform infrared (FTIR) spectral measurements on a Bruker Tensor 27 FTIR spectrometer (resolution: 4 cm^–1^).

#### DSC

The vacuum-dried self-assembled solids (3–5 mg) of pure and mixed lipids were recorded on a METTLER TOLEDO DSC882e to obtain DSC thermograms in a nitrogen atmosphere at a heating rate of 5 °C min^–1^ from 35 to 135 °C. For thermal analysis of the mechanical mixture of 18D and 16L nanotubes (18D = 16L), the dried solids of respectively pre-self-assembled 18D nanotubes (2.17 mg) and 16L nanotubes (1.98 mg) were directly added into the sample pan and measurements were performed under the same conditions as described above.

#### UV/Vis and CD

A 10^–3^ M aqueous solution of TPPS (tetrakis(4-sulfonatonphenyl)porphine, Dojindo Laboratories) was prepared and divided into several aliquots in which the as-prepared lipid gels were added. The mixtures were gently shaken for a while and settled overnight under ambient conditions for full absorption of TPPS on the surface of the nanostructures. The excess TPPS in the aqueous solutions was removed using a centrifuge (Anke TGL-16C, Shanghai) at 6000 rpm for 5 min. The green sediments were dispersed in water and re-centrifuged several times until the supernatant liquid was colourless. After that, the sediments were dispersed into 3 mL of aqueous hydrochloric acid (0.1 M) and then centrifuged to remove residual acid. Finally, the sediments were re-dispersed into methanol for UV/Vis and CD spectral measurement on a JASCO UV-550 and J-815 CD spectrophotometer, respectively.

#### MD simulation^60^–^63^

The pre-assembly aggregates of bilayers were solvated in H_2_O boxes with sufficient capacity by the PACKMOL program. Then, MD of solution systems was performed within the NPT ensemble (constant number of atoms, pressure, and temperature) in GROMACS-4.6.7. A Berendsen thermostat with a time-step of 1 fs was employed to regulate the temperature at 298 K. All simulations were carried out for 6 ns to achieve a fully relaxed configuration by using the General Amber Force-Field (GAFF).

## Conflicts of interest

There are no conflicts to declare.

## Supplementary Material

Supplementary information

## References

[cit1] Cintas P. (2002). Angew. Chem., Int. Ed..

[cit2] Perez-Garcia L., Amabilino D. B. (2007). Chem. Soc. Rev..

[cit3] Smith D. K. (2009). Chem. Soc. Rev..

[cit4] Palmans A. R. A., Meijer E. W. (2007). Angew. Chem., Int. Ed..

[cit5] Yashima E., Ousaka N., Taura D., Shimomura K., Ikai T., Maeda K. (2016). Chem. Rev..

[cit6] Pijper D., Feringa B. L. (2008). Soft Matter.

[cit7] De Feyter S., De Schryver F. C. (2003). Chem. Soc. Rev..

[cit8] Hembury G. A., Borovkov V. V., Inoue Y. (2008). Chem. Rev..

[cit9] Liu M., Zhang L., Wang T. (2015). Chem. Rev..

[cit10] Qiu H., Che S. (2011). Chem. Soc. Rev..

[cit11] Wang Y., Xu J., Wang Y., Chen H. (2013). Chem. Soc. Rev..

[cit12] Mateos-Timoneda M. A., Crego-Calama M., Reinhoudt D. N. (2004). Chem. Soc. Rev..

[cit13] Feringa B. L., van Delden R. A. (1999). Angew. Chem., Int. Ed..

[cit14] Lee S. B., Mitchell D. T., Trofin L., Nevanen T. K., Soderlund H., Martin C. R. (2002). Science.

[cit15] Lv K., Zhang L., Lu W., Liu M. (2014). ACS Appl. Mater. Interfaces.

[cit16] Sogutoglu L.-C., Steendam R. R. E., Meekes H., Vlieg E., Rutjes F. P. J. T. (2015). Chem. Soc. Rev..

[cit17] Roche C., Sun H.-J., Prendergast M. E., Leowanawat P., Partridge B. E., Heiney P. A., Araoka F., Graf R., Spiess H. W., Zeng X., Ungar G., Percec V. (2014). J. Am. Chem. Soc..

[cit18] Sato K., Itoh Y., Aida T. (2014). Chem. Sci..

[cit19] Chen T., Yang W.-H., Wang D., Wan L.-J. (2013). Nat. Commun..

[cit20] Lohr A., Wuerthner F. (2008). Angew. Chem., Int. Ed..

[cit21] Prins L. J., Huskens J., de Jong F., Timmerman P., Reinhoudt D. N. (1999). Nature.

[cit22] Arranz-Gibert P., Guixer B., Malakoutikhah M., Muttenthaler M., Guzman F., Teixido M., Giralt E. (2015). J. Am. Chem. Soc..

[cit23] Zhou J., Du X., Li J., Yamagata N., Xu B. (2015). J. Am. Chem. Soc..

[cit24] Li M., Howson S. E., Dong K., Gao N., Ren J., Scott P., Qu X. (2014). J. Am. Chem. Soc..

[cit25] Liu J., Yuan F., Ma X., Auphedeous D.-I. Y., Zhao C., Liu C., Shen C., Feng C. (2018). Angew. Chem., Int. Ed..

[cit26] Green M. M., Peterson N. C., Sato T., Teramoto A., Cook R., Lifson S. (1995). Science.

[cit27] Langeveld-Voss B. M. W., Waterval R. J. M., Janssen R. A. J., Meijer E. W. (1999). Macromolecules.

[cit28] Nonokawa R., Yashima E. (2003). J. Am. Chem. Soc..

[cit29] Oda R., Huc I., Schmutz M., Candau S. J., MacKintosh F. C. (1999). Nature.

[cit30] Jin W., Fukushima T., Niki M., Kosaka A., Ishii N., Aida T. (2005). Proc. Natl. Acad. Sci. U. S. A..

[cit31] Markvoort A. J., ten Eikelder H. M. M., Hilbers P. A. J., de Greef T. F. A., Meijer E. W. (2011). Nat. Commun..

[cit32] Cao J., Yan X., He W., Li X., Li Z., Mo Y., Liu M., Jiang Y.-B. (2017). J. Am. Chem. Soc..

[cit33] Cao H., De Feyter S. (2018). Nat. Commun..

[cit34] Kumar J., Tsumatori H., Yuasa J., Kawai T., Nakashima T. (2015). Angew. Chem., Int. Ed..

[cit35] Yamamoto T., Murakami R., Komatsu S., Suginome M. (2018). J. Am. Chem. Soc..

[cit36] Yamagishi H., Fukino T., Hashizume D., Mori T., Inoue Y., Hikima T., Takata M., Aida T. (2015). J. Am. Chem. Soc..

[cit37] Green M. M., Reidy M. P., Johnson R. J., Darling G., Oleary D. J., Willson G. (1989). J. Am. Chem. Soc..

[cit38] Palmans A. R. A., Vekemans J., Havinga E. E., Meijer E. W. (1997). Angew. Chem., Int. Ed..

[cit39] Yamasaki Y., Shio H., Amimoto T., Sekiya R., Haino T. (2018). Chem.–Eur. J..

[cit40] Ke Y.-Z., Nagata Y., Yamada T., Suginome M. (2015). Angew. Chem., Int. Ed..

[cit41] Makiguchi W., Kobayashi S., Furusho Y., Yashima E. (2013). Angew. Chem., Int. Ed..

[cit42] Nagata Y., Nishikawa T., Suginome M. (2016). ACS Macro Lett..

[cit43] Edwards W., Smith D. K. (2014). J. Am. Chem. Soc..

[cit44] Roche C., Sun H.-J., Leowanawat P., Araoka F., Partridge B. E., Peterca M., Wilson D. A., Prendergast M. E., Heiney P. A., Graf R., Spiess H. W., Zeng X., Ungar G., Percec V. (2016). Nat. Chem..

[cit45] Gulikkrzywicki T., Fouquey C., Lehn J. M. (1993). Proc. Natl. Acad. Sci. U. S. A..

[cit46] Cao H., Zhu X. F., Liu M. H. (2013). Angew. Chem., Int. Ed..

[cit47] Kawasaki T., Wakushima Y., Asahina M., Shiozawa K., Kinoshita T., Lutz F., Soai K. (2011). Chem. Commun..

[cit48] Matsumoto A., Abe T., Hara A., Tobita T., Sasagawa T., Kawasaki T., Soai K. (2015). Angew. Chem., Int. Ed..

[cit49] Singer S. J., Nicolson G. L. (1972). Science.

[cit50] Simons K., Ikonen E. (1997). Nature.

[cit51] van Meer G., Voelker D. R., Feigenson G. W. (2008). Nat. Rev. Mol. Cell Biol..

[cit52] Stals P. J. M., Artar M., Vendrig P., Palmans A. R. A., Meijer E. W. (2015). Aust. J. Chem..

[cit53] Arias S., Rodriguez R., Quinoa E., Riguera R., Freire F. (2018). J. Am. Chem. Soc..

[cit54] Cobos K., Quinoa E., Riguera R., Freire F. (2018). J. Am. Chem. Soc..

[cit55] Zhu X., Li Y., Duan P., Liu M. (2010). Chem.–Eur. J..

[cit56] Khan M. K., Sundararajan P. R. (2008). J. Phys. Chem. B.

[cit57] Ohno O., Kaizu Y., Kobayashi H. (1993). J. Chem. Phys..

[cit58] Dordevic L., Arcudi F., D'Urso A., Cacioppo M., Micali N., Burgi T., Purrello R., Prato M. (2018). Nat. Commun..

[cit59] Zhang L., Liu M. (2009). J. Phys. Chem. B.

[cit60] Martinez L., Andrade R., Birgin E. G., Martinez J. M. (2009). J. Comput. Chem..

[cit61] Hess B., Kutzner C., van der Spoel D., Lindahl E. (2008). J. Chem. Theory Comput..

[cit62] Wang J., Wang W., Kollman P. A., Case D. A. (2006). J. Mol. Graphics Modell..

[cit63] Wang J. M., Wolf R. M., Caldwell J. W., Kollman P. A., Case D. A. (2004). J. Comput. Chem..

[cit64] Selinger R. L. B., Selinger J. V., Malanoski A. P., Schnur J. M. (2004). Phys. Rev. Lett..

[cit65] Selinger J. V., Spector M. S., Schnur J. M. (2001). J. Phys. Chem. B.

