# Supplementary material for: Homochiral nanotubes from heterochiral lipid mixtures: a shorter alkyl chain dominated chiral self-assembly
Source: Chem Sci. 2019 Feb 20;10(13):3873–80. doi: 10.1039/c9sc00215d (PMC6461104; doi:10.1039/c9sc00215d)
Supplement: Supplementary file 1 [file SC-010-C9SC00215D-s001.pdf]

Electronic Supplementary Material (ESI) for Chemical Science.
This journal is © The Royal Society of Chemistry 2019

Electronic Supplementary Information

for

Homochiral Nanotubes from Heterochiral Lipid Mixtures: A Shorter Alkyl Chain Dominated Chiral Self-assembly

Xuefeng Zhu,[a] Yuqian Jiang,[b] Dong Yang,[a] Li Zhang,[a] Yuangang Li,[a] and Minghua Liu*[a,b]

[a] Beijing National Laboratory for Molecular Science (BNLMS), CAS Key Laboratory of Colloid, Interface, and Chemical Thermodynamics, Institute of Chemistry, Chinese Academy of Sciences, Beijing 100190, PR China.

[b] National Center for Nanoscience and Technology, Beijing 100190, P. R. China

[#] These authors contributed equally to this work.

[*] Corresponding author.

E-mail: liumh@iccas.ac.cn (M. H. Liu)

Table of Contents

Table of Contents 2
Experimental Procedures 3
Materials and Syntheses 3
Supplementary Figures and Tables 4
Figure S1 4
Figure S2 4
Figure S3 4
Figure S4 5
Figure S5 5
Figure S6 5
Figure S7 5
Figure S8 6
Figure S9 6
Figure S10 7
Table S1 8
Table S2 8
References 9
Author Contributions 9

Experimental Procedures

Materials and Syntheses

All the chemical materials were used as received without further treatments. The synthesis procedure has been previously reported in detail.[1] Also, N, N′-bisoctadecyl-L-amino-glutamic-diamide (18L) and N, N′-bisoctadecyl-D-amino-glutamic-diamide (18D) have been fully characterized.[1]

N, N′-bisdocosyl-L-amino-glutamic-diamide (20L): ^{1}H-NMR ($CDCl_3$, 400Hz): δ(ppm): 0.86-0.90 (t, 6H), 1.25-1.28 (m, 68H, CH_2), 1.49-1.50 (m, 4H, CH_2), 1.63 (s, 2H, NH_2), 1.95 (q, 2H, CH_2), 2.32-2.35 (m, 2H, CH_2), 3.23 (m, 4H, CH_2), 3.42 (t, 1H, CH), 6.12 (s, 1H, NH), 7.36 (s, 1H, NH). MALDI-TOF MS: calcd. for $C_{45}H_{91}N_3O_2$: m/z = 706.22; found: $[C_{45}H_{91}N_3O_2 + Na]^+$: m/z = 728.8, $[C_{45}H_{91}N_3O_2 + K]^+$: m/z = 744.7. El. Anal. calcd. for $C_{45}H_{91}N_3O_2$: C, 76.53; H, 12.99; N, 5.95; found: C, 76.06; H, 12.52; N, 6.00.

N, N′-bisdocosyl-D-amino-glutamic-diamide (20D): ^{1}H-NMR ($CDCl_3$, 400Hz): δ(ppm): 0.86-0.89 (t, 6H), 1.25-1.29 (m, 68H, CH_2), 1.49-1.51 (m, 4H, CH_2), 1.60 (s, 2H, NH_2), 1.93-1.95 (q, 2H, CH_2), 2.33 (m, 2H, CH_2), 3.25 (m, 4H, CH_2), 3.43 (t, 1H, CH), 6.15 (s, 1H, NH), 7.33 (s, 1H, NH). MALDI-TOF MS: calcd. for $C_{45}H_{91}N_3O_2$: m/z = 706.22; found: $[C_{45}H_{91}N_3O_2 + Na]^+$: m/z = 728.9, $[C_{45}H_{91}N_3O_2 + K]^+$: m/z = 744.8. El. Anal. calcd. for $C_{45}H_{91}N_3O_2$: C, 76.53; H, 12.99; N, 5.95; found: C, 76.26; H, 13.06; N, 6.02.

N, N′-bishexadecyl-L-amino-glutamic-diamide (16L): ^{1}H-NMR ($CDCl_3$, 400Hz): δ(ppm): 0.86-0.90 (t, 6H), 1.25-1.28 (m, 52H, CH_2), 1.49-1.51 (m, 4H, CH_2), 1.60 (s, 2H, NH_2), 1.93-1.97 (q, 2H, CH_2), 2.32-2.35 (m, 2H, CH_2), 3.23 (m, 4H, CH_2), 3.43 (t, 1H, CH), 6.07 (s, 1H, NH), 7.33 (s, 1H, NH). MALDI-TOF MS: calcd. for $C_{37}H_{75}N_3O_2$: m/z = 594.01; found: $C_{37}H_{75}N_3O_2$: m/z = 594.7, $[C_{37}H_{75}N_3O_2 + Na]^+$: m/z = 616.7, $[C_{37}H_{75}N_3O_2 + K]^+$: m/z = 632.7. El. Anal. calcd. for $C_{37}H_{75}N_3O_2$: C, 74.81; H, 12.73; N, 7.07; found: C, 74.76; H, 12.66; N, 6.95.

N, N′-bishexadecyl-D-amino-glutamic-diamide (16D): ^{1}H-NMR ($CDCl_3$, 400Hz): δ(ppm): 0.86-0.89 (t, 6H), 1.25-1.28 (m, 52H, CH_2), 1.47-1.50 (m, 4H, CH_2), 1.61 (s, 2H, NH_2), 1.93-1.95 (q, 2H, CH_2), 2.31-2.33 (m, 2H, CH_2), 3.20-3.26 (m, 4H, CH_2), 3.42 (t, 1H, CH), 6.19 (s, 1H, NH), 7.39 (s, 1H, NH). MALDI-TOF MS: calcd. for $C_{37}H_{75}N_3O_2$: m/z = 594.01; found: $C_{37}H_{75}N_3O_2$: m/z = 594.7, $[C_{37}H_{75}N_3O_2 + Na]^+$: m/z = 616.7. El. Anal. calcd. for $C_{37}H_{75}N_3O_2$: C, 74.81; H, 12.73; N, 7.07; found: C, 74.95; H, 12.82; N, 6.92.

N, N′-bistetradecyl-L-amino-glutamic-diamide (14L): ^{1}H-NMR ($CDCl_3$, 400Hz): δ(ppm): 0.86-0.89 (t, 6H), 1.25-1.28 (m, 44H, CH_2), 1.49-1.50 (m, 4H, CH_2), 1.93-1.97 (q, 2H, CH_2), 1.98 (br, 2H, NH_2), 2.32-2.36 (m, 2H, CH_2), 3.20-3.26 (m, 4H, CH_2), 3.45-3.48 (t, 1H, CH), 6.16 (s, 1H, NH), 7.39 (s, 1H, NH). MALDI-TOF MS: calcd. for $C_{33}H_{67}N_3O_2$: m/z = 537.91; found: $C_{33}H_{67}N_3O_2$: m/z = 538.6, $[C_{33}H_{67}N_3O_2 + Na]^+$: m/z = 560.6, $[C_{33}H_{67}N_3O_2 + K]^+$: m/z = 576.6. El. Anal. calcd. for $C_{33}H_{67}N_3O_2$: C, 73.68; H, 12.56; N, 7.81; found: C, 73.57; H, 12.62; N, 7.70.

N, N′-bistetradecyl-D-amino-glutamic-diamide (14D): ^{1}H-NMR ($CDCl_3$, 400Hz): δ(ppm): 0.86-0.89 (t, 6H), 1.26-1.28 (m, 44H, CH_2), 1.47-1.50 (m, 4H, CH_2), 1.63 (s, 2H, NH_2), 1.93-1.96 (q, 2H, CH_2), 2.31-2.33 (m, 2H, CH_2), 3.20-3.26 (m, 4H, CH_2), 3.40-3.42 (t, 1H, CH), 6.10 (s, 1H, NH), 7.33 (s, 1H, NH). MALDI-TOF MS: calcd. for $C_{33}H_{67}N_3O_2$: m/z = 537.91; found: $C_{33}H_{67}N_3O_2$: m/z = 538.6, $[C_{33}H_{67}N_3O_2 + Na]^+$: m/z = 560.6, $[C_{33}H_{67}N_3O_2 + K]^+$: m/z = 576.6. El. Anal. calcd. for $C_{33}H_{67}N_3O_2$: C, 73.68; H, 12.56; N, 7.81; found: C, 72.91; H, 12.22; N, 7.74.

Supplementary Figures and Tables

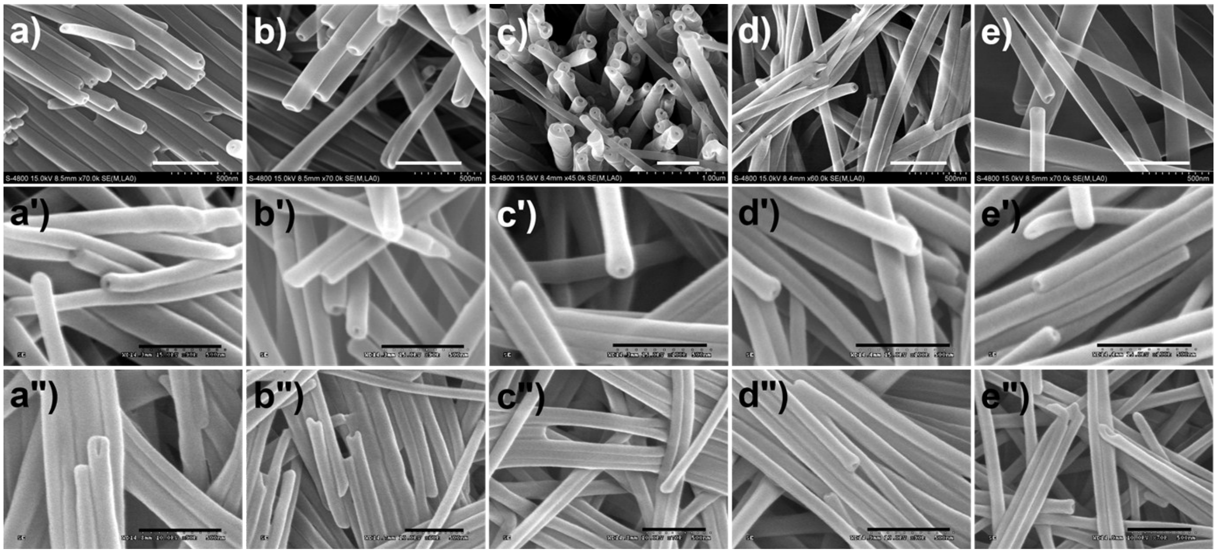

Fig. S1 High-resolution SEM images of self-assembled nanostructures from heterochiral lipids at different ratios. From left to right: D/L = 19/1, 3/1, 1/1, 1/3, 1/19. From upper to bottom: 18L/16D (a-e), 18D/16L (a' & e'), 16L/14D (a" & e"). Scale bar: 500 nm.

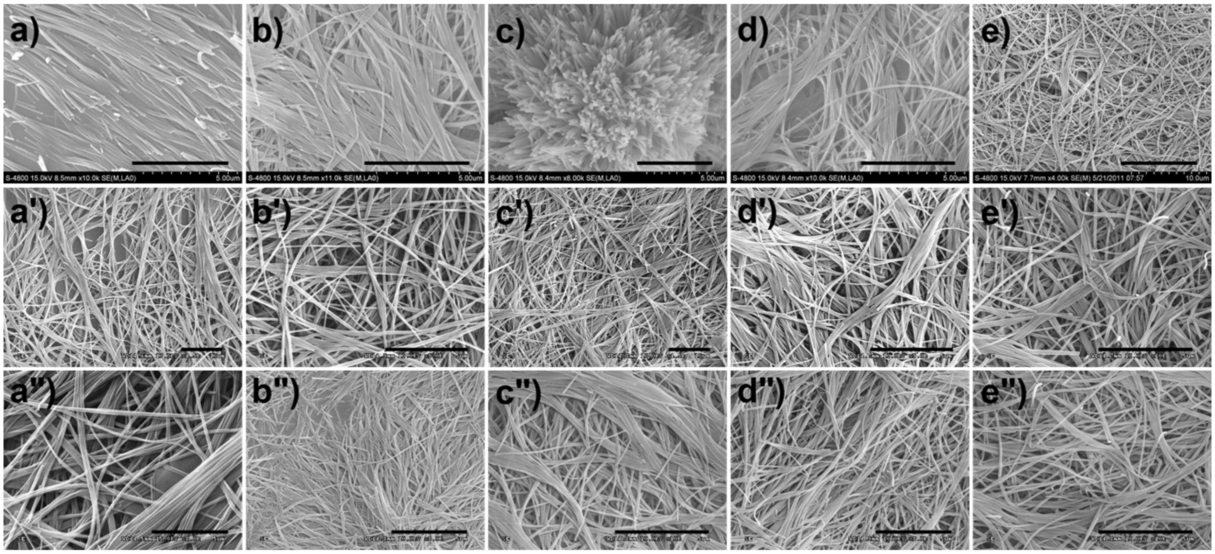

Fig. S2 Large scale SEM images of self-assembled nanostructures from heterochiral lipids at different ratios. From left to right: D/L = 19/1, 3/1, 1/1, 1/3, 1/19. From upper to bottom: 18L/16D (a-e), 18D/16L (a' & e'), 16L/14D (a" & e"). Scale bar: 5 μm.

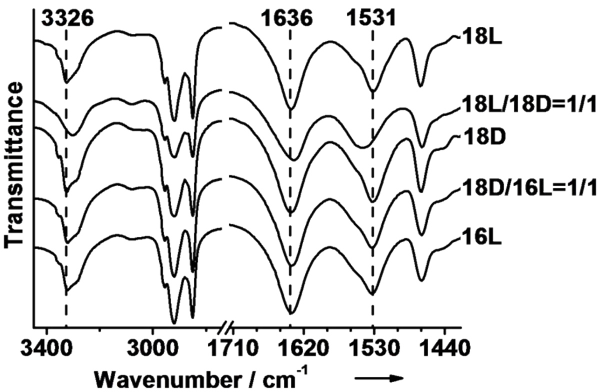

Fig. S3 FTIR spectra of self-assembled nanostructures from pure lipids and heterochiral lipids mixtures.

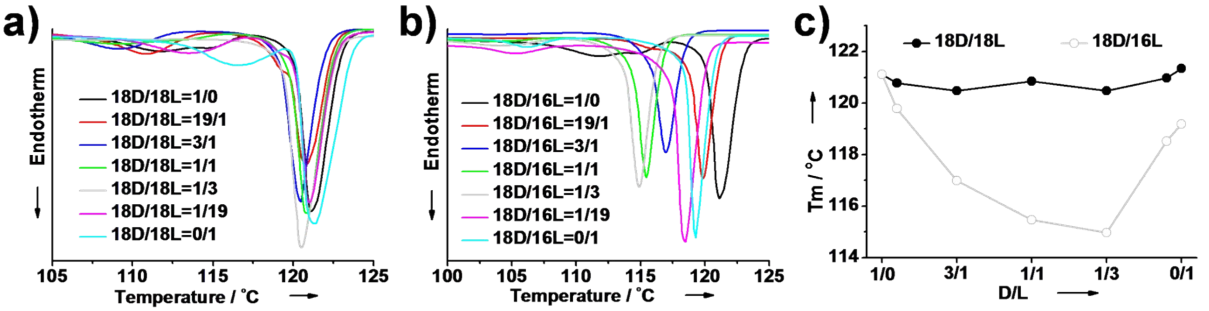

Fig. S4 DSC traces (a, b) and phase-diagram (c) of self-assembled nanostructures from pure lipids and heterochiral lipids mixtures.

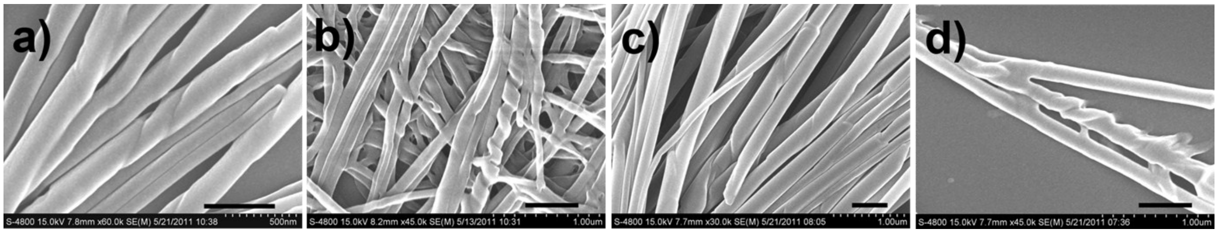

Fig. S5 SEM images of the helical nanotubes self-assembled from 18L/16D at molar ratio of a) 1.5/1, b) 3/1, c) 19/1, d) 1/3. Scale bar: 500 nm.

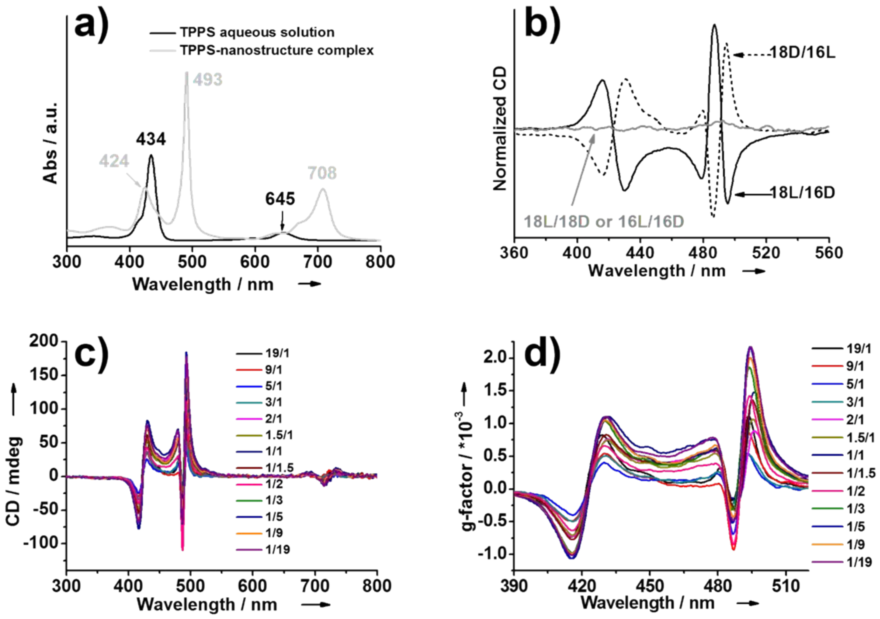

Fig. S6 UV-Vis (a), CD (b,c) and g-factor (d) spectra of TPPS aqueous solution and diverse nanostructure@TPPS complex.

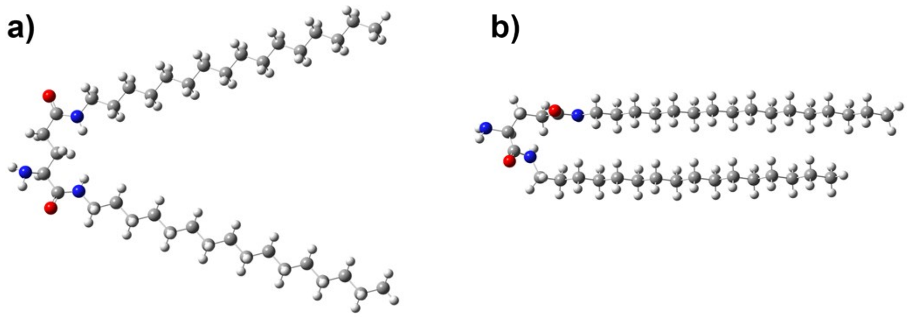

Fig. S7 Two different conformation structures of 16L molecules.

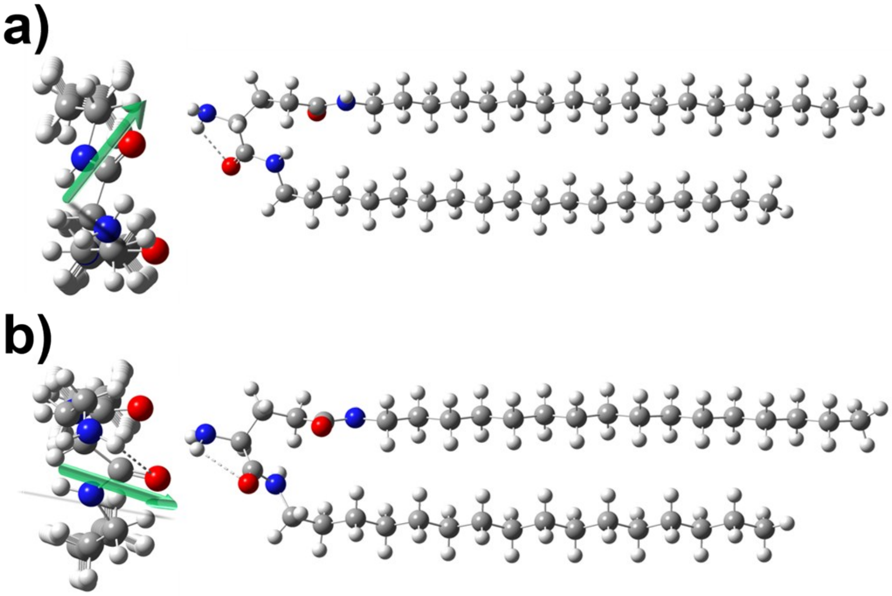

Fig. S8 The directions (d, represented by green arrows) of preferred orientation of a-amide groups in 18D (a) and 16L (b). Since there is no physical polarity along the director axis, d and -d are fully equivalent.

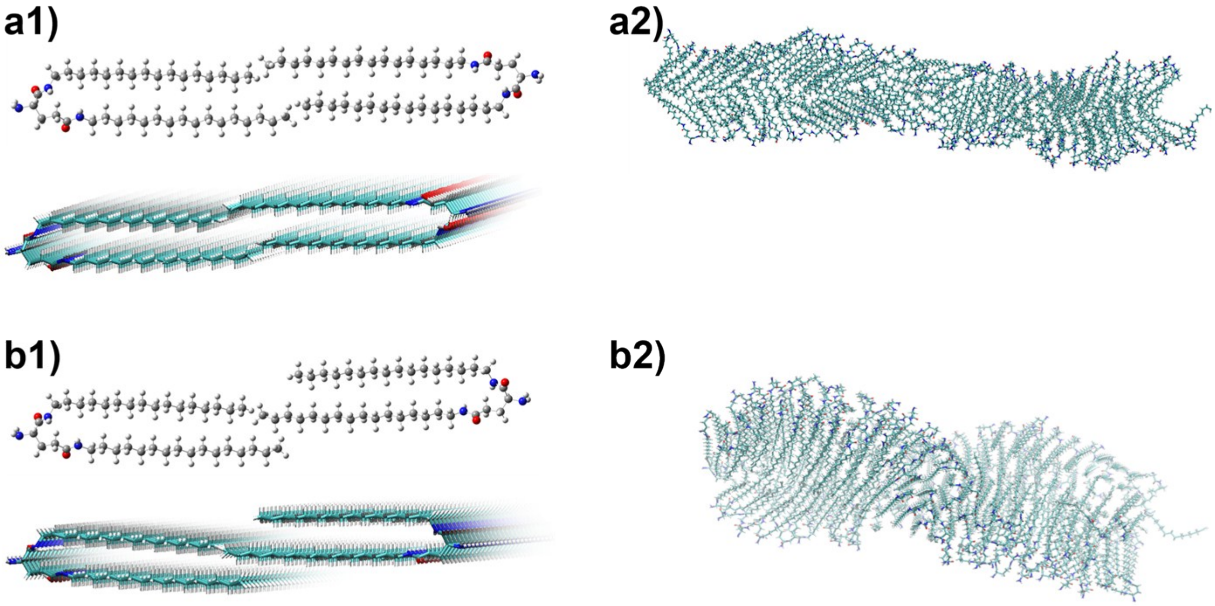

Fig. S9 Two different aggregation manners for pre-assembly 16L bilayer (a1, b1) and the resulted equilibrium configuration of 16L aggregates (a2, b2).

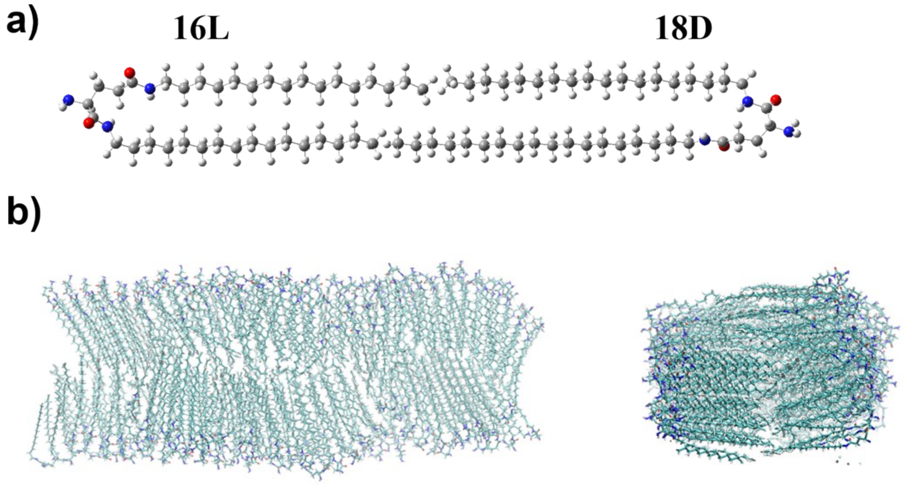

Fig. S10 The aggregation manners for pre-assembly 18D/16L combination without the conformation arrangement of 18D lipid, and the resulted equilibrium configuration of 18D/16L bilayer.

Table S1. Main assignments [cm^{-1}] of FTIR spectra of 18D/18L mixtures.

18D/18L	$\nu_{NH\&NH_2}$	$\nu(CH_2)_n$	Amide I	Amide II	δCH_2	$\delta(CH_2)_n$
1/0	3322	2920, 2851	1636	1533	1470	721
19/1	3311	2919, 2851	1633	1530	1471	720
3/1	3306	2919, 2851	1631	1543	1470	721
1/1	3302	2919, 2851	1633	1544	1469	721
1/3	3306	2919, 2850	1631	1543	1470	721
1/19	3315	2919, 2851	1633	1537	1470	721
0/1	3326	2919, 2851	1636	1532	1471	720

Table S2. Main assignments [cm^{-1}] of FTIR spectra of 18D/16L mixtures.

18D/16L	$\nu_{NH\&NH_2}$	$\nu(CH_2)_n$	Amide I	Amide II	δCH_2	$\delta(CH_2)_n$
1/0	3322	2920, 2851	1636	1533	1470	721
19/1	3321	2920, 2851	1636	1533	1470	721
3/1	3322	2920, 2851	1635	1533	1470	721
1/1	3322	2920, 2851	1635	1533	1470	721
1/3	3319	2920, 2850	1635	1534	1470	721
1/19	3323	2920, 2851	1636	1533	1470	721
0/1	3326	2919, 2851	1636	1532	1471	720

References

1 X. F. Zhu, Y. G. Li, P. F. Duan, M.H. Liu, *Chem. Eur. J.* 2010, 16, 8034-8040.
